# Assessing online gaming and pornography consumption patterns during COVID-19 isolation using an online survey: Highlighting distinct avenues of problematic internet behavior

**DOI:** 10.1016/j.addbeh.2021.107044

**Published:** 2021-12

**Authors:** Samantha N. Sallie, Valentin J.E. Ritou, Henrietta Bowden-Jones, Valerie Voon

**Affiliations:** a Department of Psychiatry, University of Cambridge, Level E4, Addenbrooke's Hospital, Cambridge CB2 0QQ, United Kingdom; b Faculty of Basic and Biomedical Sciences, University of Paris (Université de Paris), 45 rue des Saints-Pères, Paris 75006, France; c Faculty of Brain Sciences, University College London (UCL), London WC1E 6BS, United Kingdom

**Keywords:** COVID-19, Internet use, Online gaming, Pornography, Stress, Self-isolation, OG, online gaming, PV, online pornography viewing

## Abstract

**Background and aim:**

The Coronavirus (COVID-19) pandemic has required drastic safety precautions to contain virus spread, involving a protracted self-isolation period. Those with greater perceived or actual life stress are vulnerable to develop problematic internet behaviors. Thus, we assessed how COVID-19 social isolation affected online gaming (OG) and pornography viewing (PV) in the general population.

**Methods:**

We developed an online cross-sectional survey, Habit Tracker (HabiT), completed by 1,344 adults (≥18 years). HabiT was released internationally with individuals from 80 countries participating; a majority residing in the United States and United Kingdom. We measured changes in OG (IGDS9-SF) and PV (CYPAT) behaviors before and during the COVID-19 quarantine period. We also assessed psychiatric factors such as anxiety, depression (HADS), and impulsivity (SUPPS-P). The primary outcome measures were change in amount of, and current OG and PV severity during quarantine. These measures were related to ten COVID-19-related stress factors.

**Results:**

Overall, we observed a large increase in OG and a minor increase in PV. Those who increased OG (63%) and PV (43%) during quarantine were younger individuals, males, those who left the quarantine household infrequently, those who reported low frequency or poor quality social interactions, and those with higher depression, anxiety, and urgency impulsivity.

**Discussion:**

Our findings highlight similarities between forms of problematic internet behaviors driven by stress, depression, anxiety; while highlighting distinct avenues which these behaviors can manifest.

**Conclusion:**

We emphasize the relevance of identifying those in need of emotional regulation interventions, to mitigate problematic internet behaviors in the context of COVID-19 isolation.

## Highlights

• Individuals increased online gaming and online pornography viewing during quarantine.
• Those who increased these behaviors were younger individuals, males, and those reported low frequency or poor quality social interactions.
• Both groups reported higher levels of depression, anxiety, and urgency impulsivity.
• Increases in online gaming were related to changes employment and more frequent internet use.

## 1 Introduction

The Coronavirus (COVID-19) pandemic has required drastic safety precautions to contain virus spread. Although the restrictiveness of these precautions have varied widely at the country level, many countries, including the United States and United Kingdom, imposed a protracted period of self-isolation whereby individuals were neither authorized to leave their residencies unless to acquire amenities or engage in essential work, nor permitted direct contact with others beyond their immediate households. Due to these restrictions, many individuals have relied on internet use for education, work, social communication, and entertainment.

Internet use for stress relief can exert therapeutic effects when used in moderation, however, individuals with greater perceived or actual life stress are vulnerable to develop problematic internet behaviors (Ioannidis et al., 2018, Fineberg et al., 2018). These behaviors can be subdivided based on internet content, which may include gaming, gambling, social media use, pornography use, or shopping (Yellowlees and Marks, 2007, Block, 2008). The Diagnostic and Statistical Manual of Mental Disorders, Version Five (DSM-5; American Psychiatric Association, 2013) classifies Internet Gaming Disorder (IGD) under Section III as a condition requiring further research before incorporation in the main manual as a formal disorder (Potenza, 2014). Compulsive sexual behaviors, which includes problematic pornography use (PPU), has recently been accepted in the International Classification of Diseases, Eleventh Revision (ICD-11; World Health Organization, 2018) within Impulse Control Disorders (Kraus et al., 2018).

Both IGD and PPU are characterized by an excessive preoccupation with and time engaged in these activities over a prolonged period (≥6-months), often producing significant psychosocial impairment in other domains of functioning (e.g., occupational), and distress when access to gaming or pornographic material is restricted (Kim et al., 2016, Kraus et al., 2016a). The prevalence of IGD in non-pandemic conditions- varying according to characteristics of the study sample- ranges from 1–10% in Western countries (Saunders et al., 2017), while the prevalence of PPU remains contentious although estimated to occur in 5–10% of the general population (Kor et al., 2014). However, reports based on browser activity (Perez, 2020), screen time (Pantling, 2020), and search history (Pornhub, 2020) have shown that rates of both gaming and pornography consumption rose substantially coinciding with the early stay-at-home mandates (King et al., 2020, Mestre-Bach et al., 2020).

IGD and PPU have epidemiological and biological overlaps with addiction and compulsive mechanisms (Grant et al., 2010, Voon et al., 2014, Kraus et al., 2016b, Brand et al., 2016). Negative emotionality theories underlying disorders of substance addiction (Koob et al., 1998, Voon et al., 2020) may be relevant within the context of behavioral addictions and impulse control disorders, which are commonly comorbid with affective and anxiety disorders (Wang et al., 2017, Liu et al., 2018, Castro-Calvo et al., 2020). Indeed, evidence suggests depressive and anxious symptomatology have increased dramatically during the COVID-19 pandemic (Schimmenti et al., 2020, Shanahan et al., 2020), possibly as a consequence of isolation (Brooks et al., 2020, Pfefferbaum and North, 2020). Other psychiatric conditions such as pathological levels of impulsivity (Yau et al., 2012, Antons and Brand, 2018), and personality traits including high introversion and neuroticism (Müller et al., 2014, Borgogna and Aita, 2019), are often linked to IGD and PPU.

Furthermore, it has been theorized that stress can interact with these traditional vulnerability factors to produce compulsive behaviors. Acute life stress and experimental manipulations of stress trigger cue-reactivity and craving toward gaming- or pornography-related stimuli which, in turn, are related to the drive to reduce or “escape from” stress and other aversive emotions (Laier and Brand, 2017, Kaess et al., 2017); a process mediated by an impulsive coping style and positive outcome expectancies (Tonioni et al., 2014, Brand et al., 2014). Thus, the pandemic's main (e.g., risk of negative health outcomes from COVID-19 contraction) and consequent effects, such as the quarantine period and associated adverse social (e.g., lack of social interactions) and economic (e.g., change in employment status) impact, are potential stressors which may exacerbate the influence of traditional vulnerability factors on the development of problematic online behaviors.

Here, we aimed to assess how isolation measures in the midst of the COVID-19 pandemic influenced online behaviors in the general adult population. We developed an international survey, entitled Habit Tracker (HabiT), which evaluated changes in the amount and severity of these behaviors before and during the COVID-19 quarantine period. We hypothesized that the amount of online gaming (OG) and pornography viewing (PV) in our sample would be increased during the pandemic; furthermore, we hypothesized that the increase in amount and current OG and PV severity would be, in turn, related to specific COVID-19 stress factors, as well as demographic, psychiatric, and personality factors.

## 2 Methods

### 2.1 Procedure

HabiT sought to assess the effects of isolation on alcohol, smoking, and internet use. We also screened for online gambling; however, as only a limited number of respondents (n = 90) reported engaging in online gambling, we focused on the larger sample reporting OG and PV behaviors. HabiT was created using Qualtrics survey-building platform and disseminated by news agencies throughout the United Kingdom, as well as shared on social media sites from 12/05/2020 to 28/05/2020. All subjects were screened for age (18+ years) and English proficiency, and not financially compensated for participation. The average time to complete HabiT was 8–10 minutes on either a computer or smart phone. Data collected were fully anonymized. Further information regarding the creation and distribution of HabiT is found in Sallie et al. (2020).

### 2.2 Measures

#### 2.2.1 Amount and severity of OG and PV before and during quarantine

We first asked subjects if they participated in OG and PV. If the answer was negative, they proceeded to the next set of questions. If the answer was affirmative, we assessed the change in the amount and current severity of OG and PV behaviors. To assess the behavioral change before and during quarantine, we asked how many hours they spent engaging in OG and PV within the last week (i.e., during quarantine) and within a typical week in November (i.e., pre-quarantine). To assess current severity during quarantine, we used timescale-adapted versions of the Internet Gaming Disorder Scale-Short Form (IGDS9-SF; Pontes & Griffiths, 2015) and of the Cyber Pornography Addiction Test (CYPAT; Cacioppo et al., 2018). For a discussion of the psychometric properties of both scales, please refer to supplementary materials sections 1.1–1.2.

#### 2.2.2 Amount of overall internet use before and during quarantine

We assessed changes in amount of overall (non-work- or -school-related) internet use by asking participants to report how many hours on average they engaged in online activities daily both before (i.e., in November) and during (i.e., “last week”) quarantine.

#### 2.2.3 COVID-19-related stress factors

We evaluated 10 factors which may impact COVID-19-related stress with the following questions:

1. Have you been deemed an “essential worker” by your government?
• Format: Dichotomous
2. Do you work for health care services specifically with individuals who have contracted Coronavirus (COVID-19)? (Sub-question of question 1)
• Format: Dichotomous
3. Has your employment situation changed due to the Coronavirus (COVID-19) crisis?
• Format: Dichotomous
4. Has anyone you know personally contracted or have shown symptoms characteristic of Coronavirus (COVID-19)?
• Format: Dichotomous
5. Has anyone you know personally become severely ill or died due to contracting Coronavirus (COVID-19)?
• Format: Dichotomous
6. Are you isolated alone?
• Format: Dichotomous
7. Do you have children?
• Format: Dichotomous
8. If you have children, are you their only caretaker? (Sub-question of question 7)
• Format: Dichotomous
9. If you are currently in isolation with others, how would you describe the quality of your relations?
• Format: 5-point Likert (“Very uneasy,” “Somewhat uneasy,” “Neutral,” “Somewhat comfortable,” “Very comfortable”)
10. How often do you currently go outdoors (for work, essential duties, leisure, etc.)?
• Format: 5-point Likert (“Every day,” “Several times a week,” “Once a week,” “Less than once a week,” “Never”)

#### 2.2.4 Psychiatric and personality measures

Extraversion/introversion and neuroticism were assessed with two items each from the Ten-Item Personality Inventory (TIPI); a brief measure of the Big-5 personality domains (Gosling et al., 2003). Depression and anxiety symptomatology were measured using The Hospital Anxiety and Depression Scale (HADS); a validated four-item questionnaire (Snaith, 2003). Impulsivity was assessed using the Short UPPS-P Impulsive-Behavior Scale (SUPPS-P; Cyders, Littlefield, Coffey, & Karyadi, 2014). For a discussion of the psychometric properties of these scales, please refer to supplementary materials, sections 2.1.-2.3.

#### 2.2.5 Attentional checkpoints

All sections of HabiT included attentional checkpoints to ensure subjects answered survey questions to their best ability. Checkpoints were designed to reflect the Likert scaling of each section (e.g., “If you are reading this question, please select the answer choice ‘Strongly agree.’”).

#### 2.2.6 Exclusion criteria

Subjects who answered attentional checks incorrectly, reported impossible answers regarding the hours of OG and PV they engaged in weekly (i.e., over 24×7 = 168 hours), did not report their gender, nor complete the psychiatric questionnaires were excluded from analysis.

### 2.3 Statistical analysis

For a detailed description of the statistical analyses performed, please refer to Section 3 of the supplementary materials. Briefly, we utilized non-parametric tests for both OG and PV data first to compare weekly hours spent on either activity before and during quarantine (Wilcoxon-signed rank tests), and changes in weekly amount to current OG or PV severity (Kruskal-Wallis H-tests). Next, we related the ten COVID-19 stress factors to non-absolute change in weekly amount of OG and PV, and current severity of OG and PV behaviors, depression, and anxiety (Mann-Whitney U-tests and two MANCOVA analyses). Then, we assessed whether changes in OG and PV behaviors were related to one another as well as to changes in overall internet use (Spearman partial correlations). Lastly, we related current OG and PV severity to psychiatric symptoms: depression, anxiety, and impulsivity; and personality factors: extraversion and neuroticism; controlling for age and gender (Spearman partial correlations).

### 2.4 Ethics

The procedures of HabiT were in accordance with the Declaration of Helsinki and approved by the Cambridge Psychology Research Ethics Committee (approval number: PRE.2020.055). All subjects gave informed consent.

## 3 Results

### 3.1 Demographic information

A total of 2,873 subjects participated (data collection: 12/05/2020–28/05/2020) of which 1,344 had usable data based on criteria defined above in Section 2.3.6. (1,529 dropouts; 46.8% accurately completed; please refer to Section 4. of the supplementary materials for a demographic analysis of those who did not complete the survey). Of these subjects, 950 reported engaging in PV, and 746 reported engaging in OG. Of the 1,344 subjects with usable data, the average age was 28.93 ± 12.46 years (range = 18–90), with more males (males: n = 1004; females: n = 325; other: n = 15) from 80 different countries of residence, with the majority from the United Kingdom (n = 433) and the United States (n = 355). Marital status included: single: n = 785; married or committed: n = 521; divorced or separated: n = 34; widowed: n = 4. Socioeconomic status (as denoted by annual income in raw currency on the country-level) was: <19.9 k: n = 284; 20–39.9 k: n = 244; 40–69.9 k: n = 241; 70–99.9 k: n = 141; >100 k: n = 203; and 231 subjects did not report their incomes. Current psychiatric or neurological diagnoses were: no diagnosis: n = 1195; depression: n = 60; anxiety: n = 38, Post-Traumatic Stress Disorder (PTSD): n = 5, comorbid depression and anxiety: n = 46.

### 3.2 Overall changes in amount of OG and PV before versus during quarantine and severity of OG and PV

Of the total sample, the mean weekly change in amount of OG was 3.16 ± 12.46 h (range: 0–140) and PV was 0.08 ± 5.05 h, (range: 0–75). Average OG severity during quarantine was 6.74 ± 8.28, (range: 0–36), and average PV severity during quarantine was 8.55 ± 10.34, (range: 0–44), including 533 who *do not* participate in OG and 386 subjects who *do not* participate in PV.

**Fig. 1:**
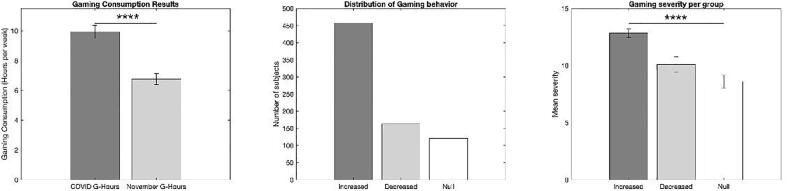
Changes in amount and current severity of online gaming (OG) behaviors in the HabiT sample between pre-quarantine and quarantine periods. Weekly hours of OG largely increased (left) and more individuals increased OG their weekly hours OG during quarantine (center). Those who increased their weekly hours of OG during the quarantine period had significantly higher OG (IGDS9-SF) severity indices (right) compared to those who decreased or did not change their weekly hours during the quarantine period. The number of asterisks in each graph represents significance level: p ≤ 0.05*, p < .005**, p < .0005***, p < .00005****.

**Fig. 2:**
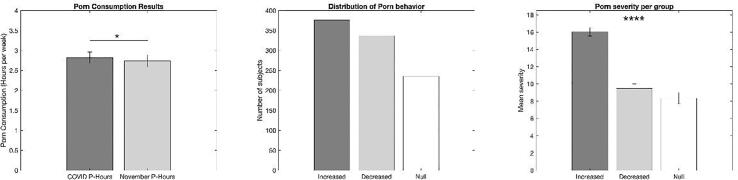
Changes in amount and current severity of pornography viewing (PV) behaviors in the HabiT sample between pre-quarantine and quarantine periods. Weekly hours of PV increased minorly, but significantly during the quarantine period (left) and more individuals either increased or decreased their weekly hours of PV during quarantine than remained the same (center). Those who increased their weekly hours of PV during the quarantine period had significantly higher PV (CYPAT) severity indices (right) compared to those who decreased or did not change their weekly hours during the quarantine period. The number of asterisks in each graph represents significance level: p ≤ 0.05*, p < .005**, p < .0005***, p < .00005****.

Of those in the sample who engage in OG (n = 771) and PV (n = 859), hours of OG per week largely increased during the quarantine period (9.92 ± 15.39 h, range = 0–120) compared to November (6.76 ± 13.2 h, range = 0–160) (W = 11.77, p < .0001) (Fig. 1), and hours of PV minorly increased during the quarantine period (2.82 ± 5.12 h, range = 0–50) compared to November (2.74 ± 5.54 h, range = 0–90) (W = 2.21, p = .03) (Fig. 2). (For changes of amount of OG and PV behaviors in the US and UK only, as well as severity of lockdown and amount of confirmed COVID-19 cases and deaths during the data collection period (Hale et al., 2020); please refer to the supplementary materials section 5.).

More subjects reported an increase (n = 485, 62.9%) as opposed to a decrease (n = 164, 21.3%) or no change (n = 122, 15.8%) of weekly hours of OG from November to quarantine (X^2^ = 35.48, p < .0001) (Fig. 1), while more subjects reported either an increase (n = 377, 44%) or a decrease (n = 337, 39%) as opposed to no change (n = 137, 17%) of weekly hours of PV from November to quarantine (X^2^ = 16.9, p < .0001) (Fig. 2).

Of the three groups, specifically, those who: 1) *increased* weekly hours during quarantine (OG: 12.57 ± 13.18 change in hours, range = 0.25–117.5; PV: 3.94 ± 4.36 change in hours, range = 0.1–40), 2) *decreased* weekly hours during quarantine (OG: −10.58 ± 15.6, range = -0.5- −140; PV: −4.1 ± 6.77, range = -0.2- −75), and 3) *did not change* their weekly hours; subjects who had increased OG and PV during the quarantine period showed significantly higher current OG and PV severity scores during quarantine (OG: 12.84 ± 7.73, range = 0–36; PV:16.1 ± 9.76, range = 0–44) than those who reported decreases (OG: 10.05 ± 8.48, range = 0–34; PV: 9.48 ± 9.8, range = 0–43) or no changes (OG: 6.16 ± 8.2, range = 0–23; PV: 8.31 ± 9.98, range = 0–40) in weekly hours (OG: H = 36.54, p < .0001; PV: H = 131.44, p < .0001) (Fig. 1, Fig. 2).

### 3.3 COVID-19 stress factor evaluation

Increased amounts of weekly OG and PV during quarantine were negatively correlated with age (OG: r_s_ = -0.43, p < .0001; PV: r_s_ = -0.29, p = .004), and males (OG: 3.35 ± 13.24 h, range = 0–140; PV: 0.093 ± 5.79 h, range = 0–75) showed a greater increase in amount of OG and PV relative to females (OG: 2.66 ± 9.82 h, range = 0–117.5; PV: 0.06 ± 1.32, range = 0–12.75) and other genders (OG: 1.50 ± 10.55, range = 0–20; PV: 0.13 ± 1.42, range = 0–3) (OG: H = 80.6, p < .0001; PV: H = 170.03, p < .0001).

Both current OG and PV severity during quarantine were also related to age and gender, with younger individuals (OG: r_s_ = -0.43, p < .0001; PV: r_s_ = -0.39, p < .0001) and males (OG: 7.94 ± 8.53, range = 0–36; PV: 10.89 ± 10.78, range = 0–44) demonstrating greater severity of these behaviors than females (OG: 3.17 ± 6.33, range = 0–34; PV: 1.69 ± 4.16, range = 0–28) and others (OG: 4.85 ± 5.9, range = 0–16; PV: 1.53 ± 2.26, range = 0–8) (OG: H = 103.81, p < .0001; PV: H = 270.42, p < .0001). Thus, age and gender were utilized as covariates for both MANCOVA analyses and dichotomized via median split (Median: age = 25 years, depression severity = 2, and anxiety severity = 1).

#### 3.3.1 Primary COVID-19 stress factors relationships with OG and PV

The influence of COVID-19 stress items on the change in weekly OG and PV amount and current OG and PV severity are reported in Table 1, Table 2, Table 3, Table 4. Changes in OG and PV amount were related to individuals not having children (OG, Table 1) and having poor quality relations with quarantine partners (PV, Table 3), respectively. However, this was no longer the case after controlling for age (OG: F = 3.62, p = .05; PV: F = 14.59, p < .0001).

**Table 1:** COVID-19 primary stress items relationship with changes in online gaming (OG) amount (in hours per week) from pre-quarantine to quarantine. Abbreviations: M = mean; SD = standard deviation; MW p-value = Mann-Whitney U-Test p-value; MAN1 p-value = MANCOVA p-value controlling for age and gender; MAN2 p-value = MANCOVA p-value controlling for age, gender, depression, and anxiety; CI = 95% confidence interval for most stringent statistically significant finding. All p-values were FDR corrected with significance assigned at p < .05. Asterisks (*) indicate a statistically significant p-value.

Stress Factor	N Total	Yes M(SD)	N Yes	No M(SD)	N No	MW p-value	MAN1 p-value	MAN2 p-value	CI
Essential worker	1269	2.45(7.8)	228	3.32(13.3)	1041	0.1	0.9	0.91	
Employment	1269	4.58(13)	302	2.72(12.3)	967	0.07	0.23	0.26	
Others ill	1266	3.26(11)	464	3.13(13.2)	802	0.87	0.9	0.91	
Others severely ill	1268	2.75(9.1)	116	3.21(12.8)	1152	0.81	0.92	0.91	
Isolated alone	1257	3.26(9.4)	159	3.17(12.9)	1098	0.87	0.9	0.91	
Having children	1266	1.45(9.6)	203	3.49(12.9)	1063	<0.0001*	0.69	0.7	0.5–3.59
Poor relationship	1109	3.5(14.2)	178	3.18(12.7)	931	0.87	0.92	0.91	
Going outdoors	1268	3.82(15)	184	3.1(12)	1084	0.66	0.9	0.91

**Table 2:** COVID-19 primary stress items relationship with current online gaming (OG) severity (i.e., timescale-adapted IGDS9-SF), depression, and anxiety from pre-quarantine to quarantine. Abbreviations: M = mean; SD = standard deviation; MW p-value = Mann-Whitney U-Test p-value; MAN1 p-value = MANCOVA p-value controlling for age and gender; MAN2 p-value = MANCOVA p-value controlling for age, gender, depression, and anxiety; CI = 95% confidence interval for most stringent statistically significant finding. All p-values were FDR corrected with significance assigned at p < .05. Asterisks (*) indicate a statistically significant p-value.

Stress Factor	N Total	Severity Type	Yes M(SD)	N Yes	N M(SD)	N No	M−W p−value	MAN1 p-value	MAN2 p-value	CI
Essential worker	1269	Gaming	5.18(7.2)	228	7.1(8.5)	1041	0.007*	0.13	0.16	
		Depression	2.27(1.8)	228	2.43(1.9)	1041	0.37	0.9		
		Anxiety	1.75(1.8)	228	1.95(1.9)	1041	0.36	0.54		
Employment change	1269	Gaming	8.16(8.7)	302	6.31(8.1)	967	0.0009*	0.004*	0.02*	−2.4 - −0.45
		Depression	2.69(2)	302	2.32(1.9)	967	0.009*	0.02*		−0.51 - −0.07
		Anxiety	2(1.9)	302	1.88(1.9)	967	0.36	0.49		
Others ill	1266	Gaming	5.4(7.6)	464	7.55(8.6)	802	<0.0001*	0.03*	0.04*	0.25–2
		Depression	2.3(1.9)	464	2.45(1.9)	802	0.23	0.79		
		Anxiety	1.87(1.8)	464	1.93(1.9)	802	0.87	0.8		
Others severely ill	1268	Gaming	5(7.6)	116	6.91(8.3)	1152	0.01*	0.21	0.24	0.45–3.45
		Depression	2.5(2)	116	2.39(1.9)	1152	0.86	0.29		
		Anxiety	1.95(2)	116	1.9(1.9)	1152	0.9	0.7		
Isolated alone	1257	Gaming	5.82(8.5)	159	6.92(8.3)	1098	0.05*	0.3	0.66	0–0.79
		Depression	2.46(2)	159	2.41(1.9)	1098	0.9	0.023*		0.46–1.02
		Anxiety	2.1(1.9)	159	1.89(1.9)	1098	0.29	0.05*		−1.24 - −0.2
Having children	1266	Gaming	1.84(4.6)	203	7.69(8.5)	1063	<0.0001*	<0.0001*	0.003*	0.98–3.52
		Depression	1.51(1.8)	203	2.58(1.9)	1063	<0.0001*	<0.0001*		2.68–2.98
		Anxiety	1.36(1.7)	203	2.02(1.9)	1063	<0.0001*	0.001*		2.2–2.55
Poor relationship	1109	Gaming	8.48(9.5)	178	6.63(8)	931	0.08	0.2	0.9	
		Depression	3.55(2)	178	2.2(1.8)	931	<0.0001*	<0.0001*		2.59–2.71
		Anxiety	2.8(2)	178	1.72(1.8)	931	<0.0001*	<0.0001*		2.14–2.51
Going outdoors	1259	Gaming	6.62(8)	184	9.66(9.4)	1084	<0.0001*	0.002*	0.02*	−2.99 - −0.59
		Depression	2.27(1.9)	184	3.3(2)	1084	<0.0001*	<0.0001*		−0.6 - −0.23
		Anxiety	1.82(1.8)	184	2.43(2)	1084	0.0002*	0.001*		−0.46 - −0.03

**Table 3:** COVID-19 primary stress items relationship with changes in amount (in hours) of online pornography viewing (PV) from pre-quarantine to quarantine. Abbreviations: M = mean; SD = standard deviation; MW p-value = Mann-Whitney U-Test p-value; MAN1 p-value = MANCOVA p-value controlling for age and gender; MAN2 p-value = MANCOVA p-value controlling for age, gender, depression, and anxiety; CI = 95% confidence interval for most stringent statistically significant finding. All p-values were FDR corrected with significance assigned at p < .05. Asterisks (*) indicate a statistically significant p-value.

Stress Factor	N Total	Yes M(SD)	N Yes	No M(SD)	N No	MW p-value	MAN1 p-value	MAN2 p-value	CI
Essential worker	1330	0.06(6.5)	240	0.1(4.7)	1090	0.68	0.94	0.98	
Employment	1330	0.12(6)	323	0.1(4.7)	1007	0.62	0.94	0.98	
Others ill	1327	0.07(3.4)	495	0.1(5.8)	832	0.39	0.94	0.98	
Others severely ill	1329	0.32(3.8)	125	0.1(5.2)	1204	0.81	0.94	0.98	
Isolated alone	1318	−0.1(8.9)	166	0.1(4.2)	1152	0.19	0.94	0.98	
Having children	1327	0.32(2.5)	208	0.04(5.4)	1119	0.62	0.94	0.98	
Poor relationship	1163	0.26(7.7)	187	-0.02(4.1)	976	0.02*	0.94	0.98	0.0–0.38
Going outdoors	1329	0.36(5.6)	189	0.04(4.9)	1140	0.2	0.94	0.98

**Table 4:** COVID-19 primary stress items relationship with current pornography viewing severity (i.e., timescale-adapted CYPAT), depression, and anxiety from pre-quarantine to quarantine. Abbreviations: M = mean; SD = standard deviation; MW p-value = Mann-Whitney U-Test p-value; MAN1 p-value = MANCOVA p-value controlling for age and gender; MAN2 p-value = MANCOVA p-value controlling for age, gender, depression, and anxiety; CI = 95% confidence interval for most stringent statistically significant finding. All p-values were FDR corrected with significance assigned at p < .05. Asterisks (*) indicate a statistically significant p-value.

Stress Factor	N Total	Severity Type	Yes M(SD)	N Yes	N M(SD)	N No	M−W p-value	MAN1 p-value	MAN2 p-value	CI
Essential worker	1330	Porn	7.38(9.9)	240	8.8(10.4)	1090	0.16	0.59	0.63	
		Depression	2.28(1.8)	240	2.42(1.9)	1090	0.45	0.86		
		Anxiety	1.78(1.8)	240	1.94(1.9)	1090	0.45	0.72		
Employment change	1330	Porn	10.5(11)	323	7.95(9.9)	1007	0.0005*	0.0004*	0.003*	−3 - −0.8
		Depression	2.71(2)	323	2.3(1.9)	1007	0.003*	0.004*		−0.31 - −0.01
		Anxiety	2.03(1.9)	323	1.87(1.8)	1007	0.3	0.34		
Others ill	1327	Porn	6.72(9.1)	495	9.7(10.8)	832	<0.0001*	0.02*	0.04*	0.27–2.26
		Depression	2.31(1.9)	495	2.46(1.9)	832	0.25	0.86		
		Anxiety	1.9(1.8)	495	1.92(1.9)	832	0.95	0.95		
Others severely ill	1329	Porn	7.8(9.9)	125	8.7(10.4)	1204	0.48	0.72	0.8	
		Depression	2.43(2)	125	2.4(1.9)	1204	0.95	0.46		
		Anxiety	1.9(1.9)	125	1.91(1.9)	1204	0.8	0.86		
Isolated alone	1318	Porn	9.49(12)	166	8.5(10.3)	1152	0.67	<0.0001*	0.0005*	−4.45 - −1.5
		Depression	2.42(2)	166	2.41(1.9)	1152	0.95	0.06		
		Anxiety	2.02(1.9)	166	1.9(1.9)	1152	0.48	0.14		
Having children	1327	Porn	2.47(5.4)	208	9.7(10.2)	1119	<0.0001*	0.0002*	0.05*	0.32–3.24
		Depression	1.51(1.8)	208	2.57(1.9)	1119	<0.0001*	<0.0001*		2.68–2.97
		Anxiety	1.39(1.7)	208	2.01(1.9)	1119	<0.0001*	0.001*		2.2–2.54
Poor relationship	1163	Porn	11.7(11)	187	7.94(9.9)	976	<0.0001*	0.004*	0.31	−3.73 - −0.85
		Depression	3.57(2)	187	2.19(1.8)	976	<0.0001*	<0.0001*		−1.53 - -0.98
		Anxiety	2.79(2)	187	1.74(1.8)	976	<0.0001*	<0.0001*		−1.3 - −7.32
Going outdoors	1329	Porn	7.89(9.9)	189	12.8(12)	1140	<0.0001*	<0.0001*	0.001*	−4.02 - −0.02
		Depression	2.27(1.9)	189	3.15(2)	1140	<0.0001*	<0.0001*		−5.61 - −0.19
		Anxiety	1.83(1.8)	189	2.42(2)	1140	0.0003	0.001*		−0.44 - 0.02

A reported change in employment status, not having a personal relationship with anyone exhibiting symptoms or diagnosed with COVID-19, and going outdoors infrequently was associated with both greater current OG (Table 2) and PV (Table 4) severity. These factors remained significantly related controlling for demographics as well as depression and anxiety. The main difference in the relationship with stress factors between the two groups was whether one was isolating alone, which was associated with PV but not OG. For individuals in the US and UK, however, isolating alone was associated with both OG and PV. Further, greater OG severity in the US and UK was associated with having a relationship with someone severely ill from COVID-19 (the opposite trend of the full sample), while PV was unrelated. (For a primary COVID-19 stress factor evaluation of US and UK data only, please refer to supplementary materials Section 6).

#### 3.3.2 Secondary COVID-19 stress factors

Two COVID-19 stress items were considered secondary as they represented a subset of a primary item. Not working for health care services (OG: U = −2, p = .05; PV: U = −2.1, p = .04) and having a second caretaker for children (OG: U = −3.11, p = .002; PV: U = −4.3, p < .0001) were both associated with a trend towards greater severity of current OG, but not when controlling for age and gender.

### 3.4 Relationships between OG and PV in COVID-19 quarantine

Controlling for age and gender, we observed a positive correlation between change in weekly amount of OG and PV from pre-quarantine to quarantine (r_s_ = 0.1, p = .03). Further, overall current OG and PV severity were positively correlated (r_s_ = 0.35, p < .0001). This relationship was even stronger for the groups which increased their weekly hours of OG and PV during quarantine (r_s_ = 0.38, p < .0001).

### 3.5 Increased amount of OG and PV in relation to increased amount of overall online activity

Controlling for age and gender, increases in OG were positively related (r_s_ = 0.27, p < .0001) to increases in overall internet use during quarantine, but not for PV.

### 3.6 Relationships of OG and PV severity during quarantine with personality and psychiatric measures

In our sample, 769 individuals completed OG (IGDS9-SF) and PV (CYPAT) severity indices. For individuals who increased use during quarantine, OG and PV severity was positively related to depression (OG: r_s_ = 0.24, p < .0001; PV: r_s_ = 0.34, p < .0001), anxiety (OG: r_s_ = 0.3, p < .0001; PV: r_s_ = 0.29, p < .0001), positive urgency (OG: r_s_ = 0.22, p < .0001; PV: r_s_ = 0.16, p = .001), and negative urgency (OG: r_s_ = 0.4, p < .0001; PV: r_s_ = 0.34, p < .0001), controlling for age and gender.

## 4 Discussion

We demonstrate a large increase in weekly amount of OG and a minor increase in weekly amount of PV from pre- to quarantine periods. Three different subpopulations were identified, with most individuals increasing OG, and either increasing or decreasing PV during quarantine. An increase in weekly hours of PV and OG during quarantine was associated with younger age, male gender, and greater OG and PV severity scores during quarantine were associated with psychological factors such as greater depression, anxiety, and mood-based impulsivity. COVID-19-related stress factors were associated with greater severity of both, including a change in employment status, not having a personal relationship with anyone diagnosed with COVID-19, and going outdoors infrequently. Isolating alone was associated with greater PV, but not OG in the overall sample; although associated with both greater OG and PV severity in our US- and UK-only sub-sample. Notably, the amount and severity of OG and PV behaviors during COVID-19 isolation were positively related and also reflected a similar pattern of relationships with COVID-related stressors, demographics, and psychiatric variables. Thus, our findings seem to underscore similarities between forms of problematic internet behaviors driven by stress. Further, we observed similar psychiatric factors, yet distinct COVID-19 stress factors, contributing to changes in both internet (i.e., isolation stressors) and alcohol use (i.e., pandemic stressors) during quarantine (Sallie et al., 2020).

OG and PV may be particularly relevant internet-based behaviors in the context of COVID-19; providing relaxing, escapist, or highly-stimulating virtual experiences that may replace the face-to-face social or sexual encounters not readily obtained during quarantine. An increase in amount of OG or PV may be related to relief of loneliness, stress, or boredom from limited social interactions (Luchetti et al., 2020, Li and Wang, 2020). Here, we show that individuals who increased their amount of weekly OG during quarantine demonstrated greater OG severity scores than those who decreased or did not change, consistent with evidence reflecting that increased OG due to stress can lead to more problematic play as characterized by urges, cravings, preoccupations, distress, and possible impairment (Ioannidis et al., 2018, Fineberg et al., 2018).

Younger individuals and males showed greater current severity and changes in weekly amount of both OG and PV during quarantine, consistent with demographic factors known to be associated with problematic OG and PV. Similarly, in studies of adults, age is commonly negatively correlated with OG and PV frequency and duration (Andreassen et al., 2016, Saunders et al., 2017, Ioannidis et al., 2018). OG also shows a male predominance (Dong et al., 2018). Males further report first exposure to pornography at a younger age, view more pornographic material in duration and amount, and use pornography more regularly for unaccompanied sexual stimulation (Hald, 2006). Males also more frequently use pornography for stress relief compared to females (Kraus et al., 2016b).

In our study, COVID-19-specific stress factors were associated with OG and PV controlling for other confounding demographic and psychiatric variables. Not having children and leaving one’s quarantine residence infrequently were related to greater severity of both OG and PV during the quarantine period. Not having children may allow individuals more time and privacy to participate in OG and PV, when controlling for potentially confounding factors of younger age and male gender. Individuals who more strictly self-isolate than others may use OG and PV to quell loneliness produced by infrequent, in-person socialization (Giardina et al., 2021). The relationship between OG and having a personal relationship with someone who exhibited severe symptoms of COVID-19 was diametrically opposed in our full versus pooled-US and -UK samples, which may reflect the high COVID-19 contraction and death rates observed in either country during the data collection period (Hale et al., 2020).

Surprisingly, individuals who changed employment status during quarantine showed greater PV severity but less OG severity, which may reflect a differential effect of how the stress of employment change or loss might influence online behavior (Kuss & Lopez-Fernandez, 2016). The interpretation of this finding is limited as we did not inquire whether the change was either positive or negative, nor the effect on financial status. Relatedly, individuals in our sample who increased overall recreational internet use during the quarantine period also increased OG, but not PV.

We further observed a positive relationship between the current severity of OG and PV and severity of psychiatric symptomology. Both positive and negative emotionality factors are shown to be associated with the development of substance- and internet-related addictions and compulsive behaviors (Koob et al., 1998, Kuss and Lopez-Fernandez, 2016, Billieux et al., 2017). Indeed, both problematic OG and PV are linked to higher levels of depression, anxiety, and urgency (Cyders and Smith, 2008, Billieux et al., 2010)- defined as a subtype of trait impulsivity reflecting the predisposition to act rashly in an intensified emotional state (Cyders & Smith, 2008). Internet use, notably, OG and PV, is often used to transiently alleviate (negative) or augment (positive) affective states (Cyders & Smith, 2008), as mediated by outcome expectancy (Brand et al., 2014).

We believe findings presented here are consistent with previous research on the relationship between stress and problematic internet behaviors, while highlighting the context-specific nature by which they may manifest. We emphasize interventions to assist vulnerable individuals in regulating aversive emotions perhaps exacerbated by the COVID-19 pandemic. Indeed, multimodal cognitive-behavioral therapies have shown efficacy in promoting adaptive coping styles to mitigate problematic internet behaviors (de Abreu and Góes, 2007, Du et al., 2010).

### 4.1 Limitations and future directions

Our study is not without limitations. A cross-sectional, retrospective survey design may be limited by sampling bias; thus, one should be cautious in drawing causal interpretations from the reported data. Relatedly, attributing direct COVID-19 lockdown effects to OG and PV severity during quarantine is not recommended, as we did not calculate a change severity index for either behavior. Also, over half of individuals did not complete the survey, possibly due to the duration. Future studies should shorten questionnaires or offer monetary incentives upon completion to mitigate non-response bias. HabiT evaluated the short-term effects of the COVID-19 pandemic on internet use; longitudinal studies are indicated to explore the potential protracted effects of COVID-19 social isolation on OG and PV.

## 5 Conclusion

Both OG and PV increased overall during lockdown, and specific groups may be at higher risk for developing problematic internet behaviors. Our findings illustrate the relevance of negative emotionality (Koob et al., 1998) and stress reduction (Kaess et al., 2017) theories to behavioral addictions and impulse control disorders. OG and PV can be performed in moderate amounts in a healthy, non-pathological manner for surrogate socialization, enjoyment, and stimulation. However, a subgroup of individuals may be at higher risk for more problematic use, and longitudinal follow-up is indicated to assess any potential extended adverse effects. The lockdown resulted in a unique array of interpersonal stressors associated with negative mental health repercussions, that may re-emerge with the enforcement of subsequent localized or national lockdowns. Our findings highlight the relevance of identifying those in need of emotional regulation interventions to mitigate problematic online behaviors in the context of COVID-19 isolation and beyond.

## 6 Data statement

All collected data and code used for analysis are available upon reasonable request.

## 7 Role of funding sources

VV is supported by a MRC Senior Clinical Fellowship (MR/P008747/1).

## CRediT authorship contribution statement

**Samantha N. Sallie:** Methodology, Investigation, Writing - original draft, Writing - review & editing, Formal analysis, Validation. **Valentin J.E. Ritou:** Data curation, Software, Visualization, Formal analysis. **Henrietta Bowden-Jones:** Conceptualization, Writing - review & editing. **Valerie Voon:** Conceptualization, Supervision, Resources, Funding acquisition, Writing - review & editing.

## Declaration of Competing Interest

The authors declare that they have no known competing financial interests or personal relationships that could have appeared to influence the work reported in this paper.
