# Supplementary material for: Assessing online gaming and pornography consumption patterns during COVID-19 isolation using an online survey: Highlighting distinct avenues of problematic internet behavior
Source: Addict Behav. 2021 Dec;123:107044. doi: 10.1016/j.addbeh.2021.107044 (PMC8434429; doi:10.1016/j.addbeh.2021.107044)
Supplement: Supplementary data 1 [file mmc1.docx]

SUPPLEMENTARY MATERIALS

1. Psychometric Properties of Online Gaming and Pornography Viewing Severity Scales

1.1. The Internet Gaming Disorder Scale-Short Form (IGDS9-SF; Pontes & Griffiths, 2015) is a 9 item short-from scale adapted from the validated 20 item Internet Gaming Disorder Scale (IGDS) which assesses problem gaming behaviors such as: a preoccupation with online gaming, experiencing negative affect from attempts to decrease online gaming, increased gaming to achieve satisfaction or pleasure, failed attempts to decrease gaming activity, loss of interest in hobbies unrelated to gaming, continued gaming despite it causing problems in other areas of one’s life, deceiving loved ones about amount of time spent gaming, using gaming to cope with or relieve a negative mood, and jeopardizing an important relationship or career opportunity due to gaming. Participants can choose between “Strongly Agree” to “Strongly Disagree” on a 5-point Likert scale. The IGDS9-SF has excellent internal consistency (Cronbach's α) of 0.96 in clinical groups.

1.2. The Cyber Pornography Addiction Test (CYPAT; Cacioppo et al., 2018) uses 11 items to assess problematic pornography viewing behaviors such as: an inability to control urges to watch pornography, neglecting partner or other loved ones to watch pornography, ignoring career or educational commitments to watch pornography, failed attempts to decrease pornography viewing, feeling like pornography is “drug-like” or addictive, continuing to watch pornography in spite of adverse consequences, watching pornography to cope with negative circumstances, using pornography to feel less isolated, loss of important relationships due to pornography viewing, watching pornography when one should be working (i.e., for either work or school), and only being capable of sexual arousal when viewing pornography. Participants can choose between “Strongly Agree” to “Strongly Disagree” on a 5-point Likert scale. The CYPAT has good internal consistency (Cronbach's α) of 0.75 in healthy groups and 0.82 in clinical groups.

2. Psychometric Properties of Personality and Psychiatric Measures

2.1 The Ten-Item Personality Inventory (TIPI; Gosling et al., 2003) is brief measure of the Big-5 personality domains. Each item consists of two adjectives using the common stem, “I see myself as:’’. Each of the five items is rated on a 7-point Likert scale ranging from “Disagree Strongly” to “Agree Strongly”. The TIPI has adequate internal consistency (Cronbach's α) of 0.68 for Extraversion and 0.5 for Neuroticism (our two personality variables of interest).

2.2 The Hospital Anxiety and Depression Scale (HADS; Snaith, 2003) is a validated four-item questionnaire which assesses anxiety (e.g., “Do you feel tense and wound up?”) and depression (e.g., “Do you take as much interest in things as you used to?”) symptomatology in clinical groups. Each item is answered on a four point (0–3) Likert scale with possible scores ranging from 0-6 for anxiety and 0-6 for depression. The internal consistency of the HADS is very good, with 0.87 for anxiety and 0.81 for depression, respectively.

2.3 The Short UPPS-P Impulsive-Behavior Scale (SUPPS-P; Cyders et al., 2014) uses 13 items to assess trait impulsivity in healthy groups. This scale provides an overall impulsivity score, as well as five scores corresponding to impulsivity subscales: perseveration, lack of premeditation, sensation-seeking, negative urgency, and positive urgency. Participants report the extent to which they agree with a statement (e.g., “When I am upset I often act without thinking”) on four-point Likert scales ranging from “Agree Strongly” to “Disagree Strongly.” Higher scores in the domains Perseverance and Premeditation indicated higher impulsivity, while for the domains Sensation Seeking, Positive, and Negative Urgency, lower scores indicate more impulsivity. The SUPPS-P has good to very good internal consistency (Cronbach's α) of 0.74-0.88 across subscales.

3. Full outline of statistical analysis for OG and PV data

Statistical analyses were performed using MATLAB (Version 2020a). Subjects who answered the attentional checks incorrectly, reported impossible answers regarding the hours of OG and PV they engaged in weekly (i.e., over 24x7=168 hours), did not report their gender, or did not complete the psychiatric questionnaires were excluded from further analysis, leaving a total of 1,344 subjects. Both gaming and pornography viewing severity scores were non-normally distributed (Shapiro-Wilk, p<.05), thus non-parametric tests were used.

We performed the same analysis procedure on both OG and PV data. First, we used Wilcoxon signed-rank tests to compare hours per week spent on either activity before and during quarantine in the full group. Then, we divided subjects into three groups, those who during quarantine either increased, decreased, or did not change their weekly hours engaging in either activity, and performed a Kruskal-Wallis H-test to compare the relative hours spent and the severity indices (as assessed by the timescale-adapted IGDS9-SF and CYPAT) of these groups.

We further assessed which COVID-19-related stress factors were related to non-absolute changes in either amount (i.e., hours engaging in OG or PV per week), current severity (i.e., timescale-adapted IGDS9-SF and CYPAT), or current depression and anxiety levels using the following tests: 1) Mann-Whitney U-Tests to compare negative versus positive responses to the COVID-19 stress items; 2) MANCOVA controlling for sex and age (MAN1); and 3) MANCOVA controlling for age, sex, depression, and anxiety symptomology (MAN2). For the MANCOVA tests, variables age, depression severity, and anxiety severity were dichotomized via median split. For the COVID-19 primary stress item comparisons (8 items), we used False Discovery Rate (FDR) to control for multiple comparisons with significance assigned at p<.05 (Benjamini & Hochberg, 1995). Questions 9 (i.e., poor relationship with quarantine partner/s) and 10 (i.e., going outdoors infrequently) were manually dichotomized for analysis; with “Very uneasy” and “Somewhat uneasy” considered a positive answer, and “Neutral,” “Somewhat comfortable,” “Very comfortable” considered a negative answer for question 9, and “Once a week,” “Less than once a week,” “Never” considered a positive answer, and “Every day” and “Several times a week” considered a negative answer for question 10. Confidence intervals (CIs) are provided with p-values for significant findings observed from the most stringent statistical test.

To assess whether OG and PV behaviors were related in our sample, we used Spearman’s partial correlations in the overall sample, controlling for age and gender, to observe the relationships between: 1) Absolute change in weekly amount of OG and PV from pre-quarantine to quarantine, and 2) Current OG and PV severity during the quarantine period.

Relatedly, to assess if changes in OG and PV behaviors were associated with changes in overall internet use, we used Spearman’s partial correlations, controlling for age and gender, between both change in OG and PV against overall online change. Further, we used two Mann-Whitney U-Tests to observe the relationship between change in individuals who increased OG (1^st^ Mann-Whitney U-Test) and PV (2^nd^ Mann-Whitney U-Test) during quarantine with those who increased overall internet use in quarantine.

In an exploratory fashion, we used Spearman’s partial correlations to compare the OG and PV severity indices of subjects who completed the timescale-adapted IGDS9-SF and CYPAT with SUPPS-P, HADS, and TIPI scores to relate OG and PV severity of the sample to psychiatric and personality measures. In order to assess a possible directional relationship in current severity of OG and PV behaviors to depression, anxiety, and impulsivity; we performed Spearman’s partial correlations with the psychiatric questionnaires for those who increased these behaviors during the quarantine period. For both correlational analyses, we controlled for age and gender, and used FDR correction for multiple comparisons.

4. Demographic Analysis of Study Drop-Outs

Although a majority of the dropout subjects (n=1,529) who entered the study provided no data (n=981), we performed a demographic analysis on dropout subjects who provided this information (n=548) to assess if those who completed the survey differed in demographic factors from those who did not. The mean age of dropout subjects was 26.58 ± 11.11 years (range= 18-80 years), significantly younger than the mean age of individuals who completed the survey (U= 3.69, *p*< .0001). Further, more males (n=387) than females (n=87) or other genders (n=7) dropped out of the study prior to completion (X^2^= 61.23, *p*< .0001).

5. Changes in OG and PV Amount from Before to During the Quarantine Period for the United Kingdom (UK) and United States (US)

5.1. UK: The hours of OG and PV per week increased between the quarantine period (OG: 13.88±15.27 hours, range= 0-120; PV: 3.45±5.33 hours, range= 0-40) and November (OG: 8.16±9.98 hours, range = 0-60; PV: 3.13±4.56 hours, range= 0-40), significantly for OG (W= 6.1, p< .0001) but not significantly for PV (W= 1.1, *p*= .29). The Oxford COVID-19 Government Response Tracker (Hale et al., 2020) indicated that the lockdown stringency index in the UK during data collection (05/12/2020 to 05/28/2020) was 88.89, with 15,684 confirmed cases and 488 deaths.

5.2. US: The hours of OG and PV per week increased between the quarantine period (OG: 17.17 ± 15.18 hours, range= 0-90; PV: 4.21 ± 5.81 hours, range= 0-35) and November (OG: 9.98±11.98 hours, range = 0-80; PV: 3.83±5.8 hours, range= 0-60), significantly for OG (W= 8.8, p< .0001) and trending towards significance for PV (W= 1.8, *p*= .06). The Oxford COVID-19 Government Response Tracker (Hale et al., 2020) indicated that the lockdown stringency index in the US during data collection (05/12/2020 to 05/28/2020) was 70.92, with 1,347,916 confirmed cases and 80,684 deaths.

6. Primary COVID-19 stress factor analysis (for UK and US data ONLY)

We conducted a separate primary COVID-19 stress factor analysis exclusively for participants residing in the US (n=355) and UK (n=433). Both MANCOVA analyses included “country of residence” as a covariate. For the UK and US only, no primary COVID stress factors were related to an increase in weekly PV amount (Table 6.1.). Increases in weekly OG amount were associated with not having children and leaving the quarantine household infrequently, although this was no longer the case when controlled for the relevant covariates (Table 6.2.). Those reporting higher levels of overall OG and PV severity during the quarantine period were more likely to have experienced employment change due to COVID-19 and not have children, controlling for all variables (Tables 6.3. & Table 6.4.). Higher OG severity, but not PV severity, was associated with infrequent departures from the quarantine household (Table 6.4.), while both higher OG and PV severities were related isolating alone, but no longer so after controlling for depressive and anxious symptomatology (Tables 6.3. & Table 6.4.).

| Stress Factor | N Total | Yes  M(SD) | N  Yes | No  M(SD) | N  No | MW  p-value | MAN1  p-value | MAN2  p-value |
| --- | --- | --- | --- | --- | --- | --- | --- | --- |
| Essential worker | 781 | 0.73(3.8) | 179 | 0.1(3.81) | 602 | 0.16 | 0.23 | 0.34 |
| Employment | 781 | 0.51(3.2) | 199 | 0.14(3.25) | 582 | 0.69 | 0.41 | 0.56 |
| Others ill | 780 | 0.16(3.2) | 350 | 0.3(3.16) | 430 | 0.53 | 0.76 | 0.88 |
| Others severely ill | 779 | 0.76(4.1) | 92 | 0.16(4.1) | 687 | 0.53 | 0.34 | 0.44 |
| Isolated alone | 772 | 0.38(6.3) | 106 | 0.32(6.31) | 666 | 0.96 | 0.23 | 0.29 |
| Having children | 779 | 0.52(2.6) | 171 | 0.15(2.57) | 608 | 0.962 | 0.41 | 0.44 |
| Poor relationship | 676 | 1(4) | 99 | 0.2(4) | 577 | 0.2 | 0.23 | 0.44 |
| Going outdoors | 779 | 0.65(3.5) | 66 | 0.2(3.5) | 713 | 0.96 | 0.47 | 0.59 |

Table 6.1. COVID-19 primary stress items relationship with changes in amount (in hours) of online pornography viewing (PV) from pre-quarantine to quarantine for the US and UK only. Abbreviations: M=mean; SD=standard deviation; MW p-value=Mann-Whitney U-Test p-value; MAN1 p-value=MANCOVA p-value controlling for age, gender, and country of residence; MAN2 p-value=MANCOVA p-value controlling for age, gender, country of residence, depression, and anxiety. All p-values were FDR corrected with significance assigned at p< .05. Asterisks (*) indicate a statistically significant finding.

| Stress Factor | N Total | Yes  M(SD) | N  Yes | No  M(SD) | N  No | MW  p-value | MAN1  p-value | MAN2  p-value |
| --- | --- | --- | --- | --- | --- | --- | --- | --- |
| Essential worker | 748 | 2.83(8) | 170 | 3.89(8) | 578 | 0.14 | 0.63 | 0.61 |
| Employment | 748 | 5(11.6) | 188 | 3.19(11.6) | 560 | 0.09 | 0.43 | 0.59 |
| Others ill | 747 | 3.29(8.3) | 330 | 3.94(8.3) | 417 | 0.9 | 0.86 | 0.85 |
| Others severely ill | 746 | 3.36(9.4) | 87 | 3.7(9.4) | 659 | 0.84 | 0.86 | 0.84 |
| Isolated alone | 739 | 3.57(9.4) | 101 | 3.7(9.4) | 638 | 0.98 | 0.62 | 0.61 |
| Having children | 746 | 1.6(10.3) | 169 | 4.25(10.3) | 577 | <.0001* | 0.85 | 0.78 |
| Poor relationship | 648 | 3.5(14.3) | 96 | 3.8(14.4) | 552 | 0.98 | 0.8 | 0.67 |
| Going outdoors | 746 | 5.9(11.3) | 66 | 3.44(11.3) | 680 | 0.04* | 0.37 | 0.58 |

Table 6.2. COVID-19 primary stress items relationship with changes in online gaming (OG) amount (in hours per week) from pre-quarantine to quarantine for the US and UK only. Abbreviations: M=mean; SD=standard deviation; MW p-value=Mann-Whitney U-Test p-value; MAN1 p-value=MANCOVA p-value controlling for age, gender, and country of residence; MAN2 p-value=MANCOVA p-value controlling for age, gender, country of residence, depression, and anxiety. All p-values were FDR corrected with significance assigned at p< .05. Asterisks (*) indicate a statistically significant finding.

| Stress Factor | N  Total | Severity Type | Yes  M(SD) | N  Yes | N  M(SD) | N  No | M-W  p-value | MAN1  p-value | MAN2  p-value |
| --- | --- | --- | --- | --- | --- | --- | --- | --- | --- |
| Essential worker | 748 | Porn | 7.38(9.9) | 179 | 8.8(10.4) | 602 | 0.97 | 0.99 | 0.63 |
|  |  | Depression | 2.28(1.8) | 179 | 2.42(1.9) | 602 | 0.98 | 0.74 |  |
|  |  | Anxiety | 1.78(1.8) | 179 | 1.94(1.9) | 602 | 0.42 | 0.44 |  |
| Employment change | 748 | Porn | 10.5(11) | 199 | 7.95(9.9) | 582 | .004* | .02* | .03* |
|  |  | Depression | 2.71(2) | 199 | 2.3(1.9) | 582 | .03* | 0.18 |  |
|  |  | Anxiety | 2.03(1.9) | 199 | 1.87(1.8) | 582 | 0.42 | 0.74 |  |
| Others ill | 747 | Porn | 6.72(9.1) | 350 | 9.7(10.8) | 430 | 0.16 | 0.74 | 0.42 |
|  |  | Depression | 2.31(1.9) | 350 | 2.46(1.9) | 430 | 0.97 | 0.74 |  |
|  |  | Anxiety | 1.9(1.8) | 350 | 1.92(1.9) | 430 | 0.91 | 0.83 |  |
| Others severely ill | 746 | Porn | 7.8(9.9) | 92 | 8.7(10.4) | 687 | 0.44 | 0.58 | 0.8 |
|  |  | Depression | 2.43(2) | 92 | 2.4(1.9) | 687 | 0.98 | 0.47 |  |
|  |  | Anxiety | 1.9(1.9) | 92 | 1.91(1.9) | 687 | 0.97 | 0.74 |  |
| Isolated alone | 739 | Porn | 9.49(12) | 106 | 8.5(10.3) | 666 | .01* | .05* | 0.47 |
|  |  | Depression | 2.42(2) | 106 | 2.41(1.9) | 666 | 0.82 | 0.3 |  |
|  |  | Anxiety | 2.02(1.9) | 106 | 1.9(1.9) | 666 | 0.97 | 0.36 |  |
| Having children | 746 | Porn | 2.47(5.4) | 171 | 9.7(10.2) | 608 | <.0001* | .01* | .05* |
|  |  | Depression | 1.51(1.8) | 171 | 2.57(1.9) | 608 | <.0001* | 0.18 |  |
|  |  | Anxiety | 1.39(1.7) | 171 | 2.01(1.9) | 608 | .0002* | 0.18 |  |
| Poor relationship | 648 | Porn | 11.7(11) | 99 | 7.94(9.9) | 577 | 0.08 | 0.33 | 0.81 |
|  |  | Depression | 3.57(2) | 99 | 2.19(1.8) | 577 | <.0001* | <.0001* |  |
|  |  | Anxiety | 2.79(2) | 99 | 1.74(1.8) | 577 | <.0001* | <.0001* |  |
| Going outdoors | 746 | Porn | 7.89(9.9) | 66 | 12.8(12) | 713 | .02* | 0.16 | 0.41 |
|  |  | Depression | 2.27(1.9) | 66 | 3.15(2) | 713 | .0002* | .0009* |  |
|  |  | Anxiety | 1.83(1.8) | 66 | 2.42(2) | 713 | 0.15 | 0.22 |  |

Table 6.3. COVID-19 primary stress items relationship with current pornography viewing severity (i.e., timescale-adapted CYPAT), depression, and anxiety from pre-quarantine to quarantine for the US and UK only. Abbreviations: M=mean; SD=standard deviation; MW p-value=Mann-Whitney U-Test p-value; MAN1 p-value=MANCOVA p-value controlling for age, gender, and country of residence; MAN2 p-value=MANCOVA p-value controlling for age, gender, country of residence, depression, and anxiety. All p-values were FDR corrected with significance assigned at p< .05. Asterisks (*) indicate a statistically significant finding.

| Stress Factor | N  Total | Severity Type | Yes  M(SD) | N  Yes | N  M(SD) | N  No | M-W  p-value | MAN1  p-value | MAN2  p-value |
| --- | --- | --- | --- | --- | --- | --- | --- | --- | --- |
| Essential worker | 748 | Gaming | 4.8(8) | 170 | 3.9(8.02) | 578 | 0.07 | 0.09 | 0.2 |
|  |  | Depression | 2.2(1.8) | 170 | 2.2(1.8) | 578 | 0.97 | 0.73 |  |
|  |  | Anxiety | 1.6(1.68) | 170 | 1.9(1.68) | 578 | 0.32 | 0.26 |  |
| Employment change | 748 | Gaming | 8(11.6) | 188 | 3.2(11.6) | 560 | .0005* | 0.13 | .05* |
|  |  | Depression | 2.5(2.02) | 188 | 2.1(2.02) | 560 | 0.032 | 0.17 |  |
|  |  | Anxiety | 1.9(1.89) | 188 | 1.8(1.89) | 560 | 0.58 | 0.96 |  |
| Others ill | 747 | Gaming | 5.1(8.34) | 330 | 3.9(8.34) | 417 | .003* | 0.18 | 0.2 |
|  |  | Depression | 2.2(1.81) | 330 | 2.2(1.93) | 417 | 0.87 | 0.79 |  |
|  |  | Anxiety | 1.8(9.43) | 330 | 1.9(1.81) | 417 | 0.98 | 0.89 |  |
| Others severely ill | 746 | Gaming | 4.3(2.13) | 87 | 3.7(9.43) | 659 | .02* | 0.44 | 0.42 |
|  |  | Depression | 2.4(2) | 87 | 2.2(2.13) | 659 | 0.8 | 0.19 |  |
|  |  | Anxiety | 1.9(9.41) | 87 | 1.9(2.01) | 659 | 0.98 | 0.55 |  |
| Isolated alone | 739 | Gaming | 5.2(2.08) | 101 | 3.7(9.41) | 638 | .01* | 0.29 | 0.41 |
|  |  | Depression | 2.2(2.02) | 101 | 2.2(2.08) | 638 | 0.87 | 0.18 |  |
|  |  | Anxiety | 2(10.3) | 101 | 1.8(2.02) | 638 | 0.79 | 0.15 |  |
| Having children | 746 | Gaming | 1.7(1.91) | 169 | 4.3(10.3) | 577 | <.0001* | .01* | .05* |
|  |  | Depression | 1.6(1.81) | 169 | 2.4(1.91) | 577 | <.0001* | 0.13 |  |
|  |  | Anxiety | 1.4(1.81) | 169 | 2(1.81) | 577 | .0001* | 0.15 |  |
| Poor relationship | 648 | Gaming | 8.4(14.3) | 96 | 3.8(14.3) | 552 | 0.1 | 0.13 | 0.3 |
|  |  | Depression | 3.4(2.12) | 96 | 2(2.12) | 552 | <.0001* | <.0001* |  |
|  |  | Anxiety | 2.9(2.11) | 96 | 1.7(2.11) | 552 | <.0001* | <.0001* |  |
| Going outdoors | 746 | Gaming | 9.3(11.3) | 66 | 3.4(11.3) | 680 | .005* | .008* | .05* |
|  |  | Depression | 3.2(2.04) | 66 | 2.1(2.04) | 680 | .0001* | .001* |  |
|  |  | Anxiety | 2.3(2.05) | 66 | 1.8(2.05) | 680 | 0.1 | 0.17 |  |

Table 2. COVID-19 primary stress items relationship with current online gaming (OG) severity (i.e., timescale-adapted IGDS9-SF), depression, and anxiety from pre-quarantine to quarantine. Abbreviations: M=mean; SD=standard deviation; MW p-value=Mann-Whitney U-Test p-value; MAN1 p-value=MANCOVA p-value controlling for age, gender, country of residence; MAN2 p-value=MANCOVA p-value controlling for age, gender, country of residence, depression, and anxiety. All p-values were FDR corrected with significance assigned at p< .05. Asterisks (*) indicate a statistically significant finding.
